# A molecular phylogeny of Alpine subterranean Trechini (Coleoptera: Carabidae)

**DOI:** 10.1186/1471-2148-13-248

**Published:** 2013-11-13

**Authors:** Arnaud Faille, Achille Casale, Michael Balke, Ignacio Ribera

**Affiliations:** 1 Zoologische Staatsammlung, Muenchhausenstrasse 21, Munich 81247, Germany; 2 Dipartimento di Scienze della Natura e del Territorio, Sez. Zoologia, Università di Sassari, Via Muroni 25, Sassari 07100, Italy; 3 Institute of Evolutionary Biology (CSIC-Universitat Pompeu Fabra), Passeig Maritim de la Barceloneta 37-49, Barcelona 08003, Spain

## Abstract

**Background:**

The Alpine region harbours one of the most diverse subterranean faunas in the world, with many species showing extreme morphological modifications. The ground beetles of tribe Trechini (Coleoptera, Carabidae) are among the best studied and widespread groups with abundance of troglobionts, but their origin and evolution is largely unknown.

**Results:**

We sequenced 3.4 Kb of mitochondrial (*cox1*, *rrnL*, *trnL*, *nad1*) and nuclear (*SSU*, *LSU*) genes of 207 specimens of 173 mostly Alpine species, including examples of all subterranean genera but two plus a representation of epigean taxa. We applied Bayesian methods and maximum likelihood to reconstruct the topology and to estimate divergence times using a priori rates obtained for a related ground beetle genus. We found three main clades of late Eocene-early Oligocene origin: (1) the genus *Doderotrechus* and relatives; (2) the genus *Trechus sensu lato*, with most anisotopic subterranean genera, including the Pyrenean lineage and taxa from the Dinaric Alps; and (3) the genus *Duvalius sensu lato*, diversifying during the late Miocene and including all subterranean isotopic taxa. Most of the subterranean genera had an independent origin and were related to epigean taxa of the same geographical area, but there were three large monophyletic clades of exclusively subterranean species: the Pyrenean lineage, a lineage including subterranean taxa from the eastern Alps and the Dinarides, and the genus *Anophthalmus* from the northeastern Alps. Many lineages have developed similar phenotypes independently, showing extensive morphological convergence or parallelism.

**Conclusions:**

The Alpine Trechini do not form a homogeneous fauna, in contrast with the Pyrenees, and show a complex scenario of multiple colonisations of the subterranean environment at different geological periods and through different processes. Examples go from populations of an epigean widespread species going underground with little morphological modifications to ancient, geographically widespread lineages of exclusively subterranean species likely to have diversified once fully adapted to the subterranean environment.

## Background

Different Arthropod lineages have successfully colonised subterranean environments, prevalent among them are aquatic and terrestrial crustaceans, arachnids, myriapods and insects, in particular Coleoptera [1]. In the western Palaearctic, two groups of Coleoptera include the majority of the underground diversity: the tribes Trechini (family Carabidae) and Leptodirini (family Leiodidae), with hundreds of species with different degrees of morphological modifications assumed to be adaptations to the subterranean environment – different degrees of “troglomorphism” [2-4]. The high degree of parallelism or convergence in these modifications has always been an added difficulty to reveal phylogenetic relationships among the subterranean species [5-8], but at the same time poses a very interesting evolutionary problem, as the mechanisms through which it is achieved remain largely unknown.

According to the traditional view, virtually every species of subterranean Coleoptera developed the troglomorphic characters independently, but recent work on the Pyrenean fauna has challenged this assumption by demonstrating a single origin for ancient and diverse clades of exclusively subterranean species [9,10]. At the same time, the phylogeny of Pyrenean Trechini shows that the currently recognised genera (*Aphaenops* (*nomen protectum*[11]), *Hydraphaenops* and *Geotrechus*), which were recognised mainly based on strong similarities in the general body shape [9,10,12], are largely poly- or paraphyletic, with a striking degree of morphological convergence.

In a wider study, Faille et al. [13] included other highly modified western Mediterranean cave Trechini considered by some authors to be related to the Pyrenean fauna (i.e. to be members of the “phyletic lineage” of *Aphaenops* in the sense of Jeannel [2]), such as the Iberian *Apoduvalius* and *Paraphaenops*, *Speotrechus* from southeastern France, or the Sardinian *Sardaphaenops*. All these species were found to be not related to the Pyrenean fauna but to other lineages in the same geographical areas, showing again a strong morphological convergence among them [13]. However, in that study the basal relationships among the main lineages of the western Mediterranean fauna of Trechini could not be established, likely due to the lack of representation of more eastern lineages.

Here we extend the study of subterranean Trechini to the fauna of the Alps, which include members of the *Aphaenops* lineage assumed to be related to the Pyrenean fauna but also members of the traditionally recognised second major division of Trechini, the “isotopics” (in opposition to the “anisotopics”, which include all the species of the *Aphaenops* lineage). These two divisions refer to morphological characters of the male genitalia (see below). The fauna of the Alps is extremely rich in both epigean (i.e., living “above ground”, in the surface) and hypogean (i.e. living “below ground”) Carabidae, with more than 650 species in the eastern part –approximately half of the Italian ground beetle fauna, and a fifth of all European species, from the Canary Islands to the Urals. This area includes more than 200 endemic species, most of them concentrated on the eastern pre-Alpine belt (the “Southern Alps”, from the Como lake to Trieste) and many of them subterranean [14]. It is considered a hotspot of subterranean biodiversity, comparable to the Dinaric Alps or the Pyrenean chain [15]. The easternmost area of the Alps (the “Suprapannonian sector”sensu Ozenda & Borel [16], around Graz) lacks limestone formations and is less rich in subterranean species (Additional file 1: Figure S1). Similarly, the western part of the chain, from the Lepontine to the Maritime-Ligurian Alps, is poor in subterranean species due to the scarcity of limestone in the area north of Torino [17], despite having a very diverse fauna, with 440 species of Carabidae and a high number – c. 30% – of endemics [18]. Only the Ligurian Alps are entirely calcareous and markedly karstified, with deep and large subterranean systems even at high altitude [19] (Additional file 1: Figure S1).

The Trechini fauna of the Alpine range is one of the best known in the world due to two centuries of studies by professional and amateur speleologists and biospeleologists. The subterranean species are currently grouped into 14 genera, 13 of them endemic to the Alps [11,20-22]. The origin of this subterranean fauna has been debated at length by different authors, who proposed contrasting hypotheses [2,14,18-20]. Some of the most specialised and emblematic species have been related to the Pyrenean fauna due to their strikingly similar external appearance, in particular some ultra-specialized Trechini very similar to the Pyrenean *Aphaenops* (the so called “aphaenopsian” shape [1,23]). Within the Alps, some genera have fragmented distributions, such as *Duvalius* or *Trichaphaenops*, or morphologically similar genera occupy isolated areas, such as *Boldoriella* and *Orotrechus*, and yet some others have divergent morphologies with unknown affinities, such as *Doderotrechus*, *Italaphaenops*, *Allegrettia* or *Lessinodytes*[14,18-20,24-27]. With this study we attempt to clarify the geographical and temporal origin of all these subterranean taxa with the use of molecular data and a comprehensive representation of the subterranean and epigean European Trechini, including a wide sample of the most diverse and widespread genera (*Trechus* and *Duvalius*) as well as examples of all Alpine troglobiont genera but two. Using c. 4Kb of a combination of mitochondrial and nuclear markers of 207 specimens in 173 species we use maximum likelihood and Bayesian methods to build a calibrated phylogeny to estimate the temporal origin and the relationships of the subterranean taxa, with a special interest in the most morphologically deviant species, showing the highest degree of troglomorphism and the most enigmatic origin.

### Taxonomic background of the Alpine Trechini

Trechini is a highly diverse and cosmopolitan tribe of carabid beetles, divided in well characterised subtribes [22] (see [28] for an overview of the phylogenetic placement of Trechini). All subterranean European species, together with their related epigean groups, belong to subtribe Trechina, which are traditionally divided into two groups of genera on the basis of differences in the male genitalia. Thus, in the isotopics the endophallus has a symmetric copulatory piece in ventral position (“série phylétique de *Duvalius*” sensu Jeannel, 1928 [2]), while in the anisotopics the copulatory piece is asymmetric and placed in lateral position (Additional file 2: Figure S2) [6].

**Figure 1:**
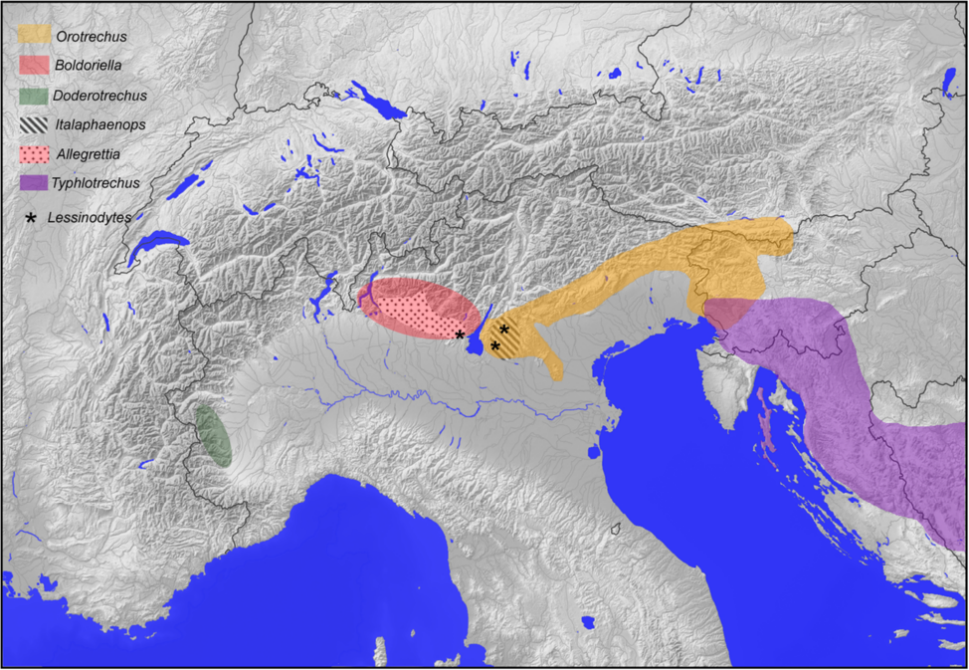
**Simplified map of the distribution of the anisotopic genera of Trechina in the Alpine region (the widespread genus *Trechus *is not represented).** See Additional file 5: Table S2 and Additional file 4: Figure S3 for details of the sampled localities.

There are nine anisotopic and eight isotopic genera of Trechina in the Alpine chain (see Additional file 3: Table S1 for a list of genera, their distribution and number of species, and the number of specimens included in our analyses). Seven of the anisotopic genera are found in the southeastern part of the chain, from the Como Lake (Lombardy, Italy) to Trieste and Slovenia (Figure 1). The only exceptions are *Doderotrechus*, an endemic to the Cottian Alps in the Italian Piedmont living in natural limestone caves, artificial mines and the MSS (“millieu souterrain superficiel”, or mesovoid shallow substratum), and the diverse, widespread and mostly epigean genus *Trechus*. *Trechus* has more than 800 species, mostly in the Palearctic region [12,22], and includes widespread winged species as well as short-range wingless endemics, many of them restricted to individual mountain massifs [29-31]. There are many species with different degrees of troglomorphism. Faille et al. [9,13] shown that the genus is a paraphyletic assemblage with some troglobitic genera or species nested within it. In the Alpine chain there are no species of *Trechus* exclusive of the subterranean environment, although some species are regularly found in it, and some high altitude orophilous species (e.g. in the *T. strigipennis* group) show depigmentation and reduced eyes.

**Figure 2:**
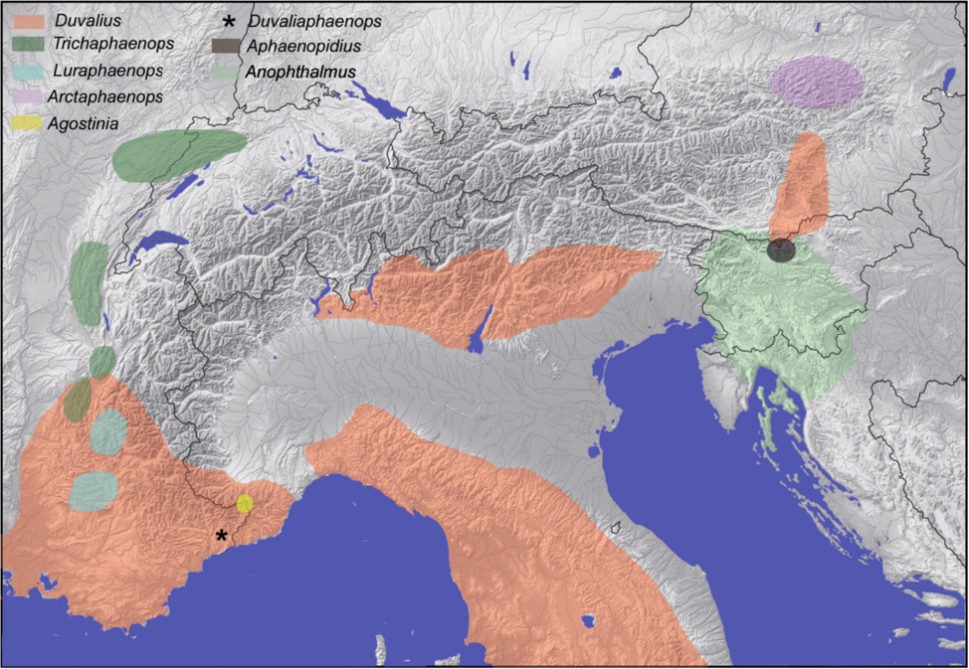
**Simplified map of the distribution of the isotopic genera of Trechina in the Alpine region.** See Additional file 5: Table S2 and Additional file 4: Figure S3 for details of the sampled localities.

Seven of the eight isotopic genera of Trechina present in the Alps are subterranean and endemic to this area (Additional file 3: Table S1). Only one of them, *Duvalius*, has a wider distribution (Figure 2). The ecological preferences and degree of troglomorphism of the members of the speciose genus *Duvalius* are very diverse: some are depigmented and blind, hygrophilous, nivicolous and living in caves, deep soil or MSS; but others are epigean, living in forest litter and alpine pastures, and are slightly pigmented and/or with reduced eyes, and even (albeit exceptionally) winged [32]. The genus is widely distributed from Spain, Maghreb (Algeria) and France in the West to central Asia and China in the East. It is diversified mostly in the Alps, Italian and Balkan peninsulas and the Carpathian region, with some species in Catalonia, Mallorca, Sicily and Sardinia. Easter of the Alps the genus is recorded from the Caucasus, Middle East and Iran, reaching the Tien Shan Mountains in China [12]. The Alpine species belong to the subgenus *Duvalius* and have been divided into various species groups (14 for the Italian species [20]), which in turn have been divided in more numerous “lineages” [33,34], mostly based on the structure of the copulatory piece. The genus was considered as a complex by Jeannel [23], with more troglomorphic genera derived from local species pools.

### Other subterranean Trechini potentially related to the Alpine fauna

We included in our study three species of the two anisotopic genera *Neotrechus* and *Adriaphaenops* from the Dinaric Alps, to test for potential relationships with the species of the Alpine fauna. The Dinaric karst is a recognised hotspot of subterranean biodiversity [35] especially rich in highly specialised troglomorphic Trechini, with new genera and species described every year (see e.g. [36,37]). The phylogenetic relationships of this highly diverse trechine fauna are unknown, but the two genera included here have been related to Alpine or Pyrenean taxa: *Neotrechus* is currently considered as close to the Alpine genus *Orotrechus*, whereas *Adriaphaenops* was considered a representative of the former *Aphaenops* lineage. Nevertheless, recent molecular results cast doubts on the possibility of such relationships [9,38]. We also include an example of *Pheggomisetes* (with three described species), known from some caves in the Bulgarian and Serbian Balkans and with a highly derived morphology. It was considered as an isolated relict of the Eocene fauna, without clear affinities with the extant Trechini [2].

## Results

### Phylogenetic analysis

**Figure 3:**
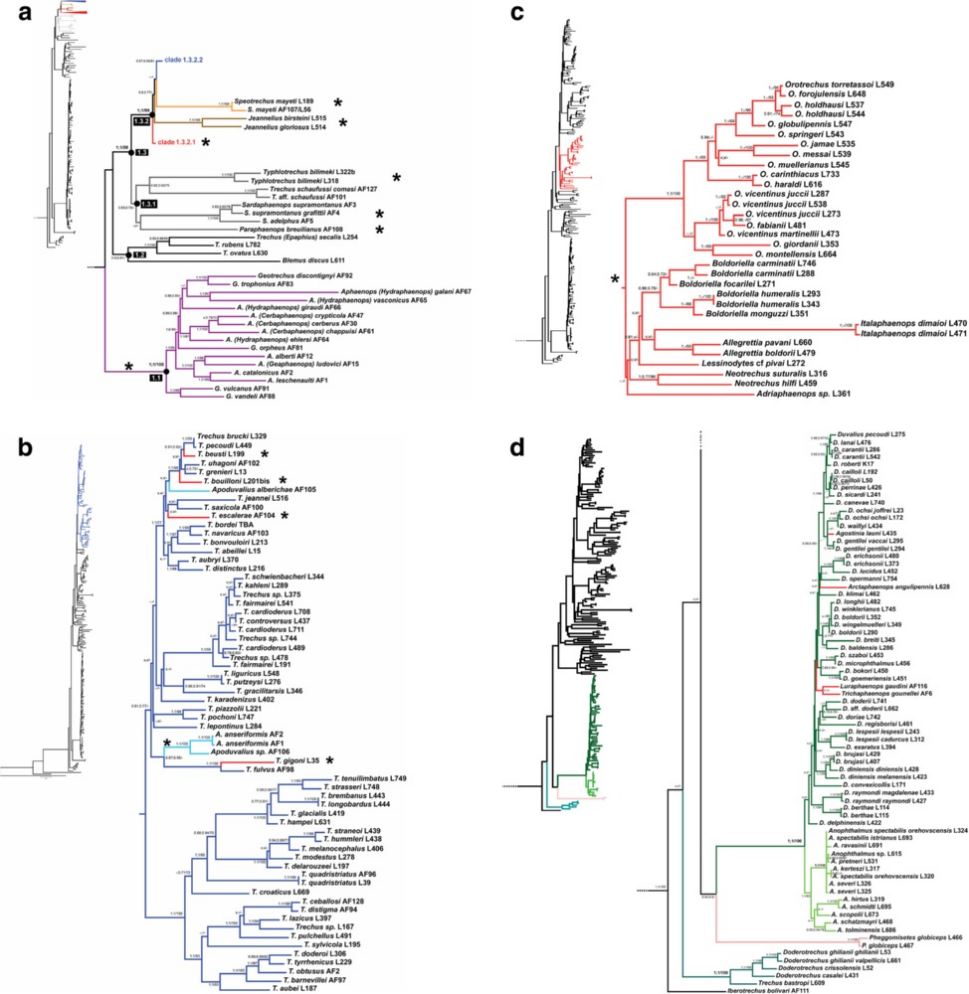
**Phylogram of Alpine Trechina obtained in RAxML using the combined data matrix.** Number in nodes, Bayesian posterior probability obtained in the two alternative states of MrBayes (see main text) a⁄ ML bootstrap obtained in RAxML. Subterranean genera and lineages are indicated by asterisks (see Additional file 3: Table S1). -: unresolved or poorly supported node (bt ≤ 70%, Bpp ≤ 70); x: contradictory node. 3a: clade 1; 3b: clade 1.3.2.2; 3c: clade 1.3.2.1; 3d: clades 2 and 3.

The estimated optimal evolutionary model was GTR + I + G for the three mitochondrial gene partitions (*cox1*, *rrnL* + *trnL* and *nad1*) and the nuclear small ribosomal unit (*SSU*), and GTR + G for the nuclear large ribosomal unit (*LSU*). In each of two independent MrBayes analyses the two runs (i.e. a total of four runs) reached convergence after 34 MY generations as measured with the effective sample size (ESS). The combination of the four runs also converged for most of the parameters (including the total likelihood of the trees), but had two alternative states (with two runs each) differing substantially in some parameters that did not converge, such as e.g. the total branch length, double in one state than in the other, or the alpha parameter of the gamma distribution in all partitions except the nuclear *LSU*. Despite the large differences in some of the parameters the two topologies were congruent for all well supported nodes (Figure 3). The topology obtained with Maximum Likelihood in RAxML was also generally congruent with the MrBayes trees, with only some poorly supported nodes showing incongruence (Figure 3) (see detailed comments below).

**Figure 4:**
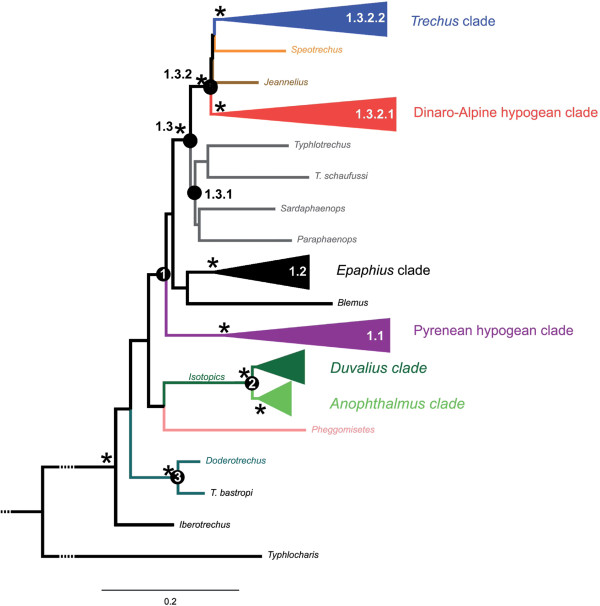
**Summary tree with the main clades of Alpine Trechini, as obtained with RAxML.** Stars, nodes with a support of pp = 1 for both Bayesian topologies and bt = 100 for RAxML. -, unresolved or poorly supported node (bt ≤ 70%, Bpp ≤ 70); x, node not present. Node labels refer to major clades (see text and Figure 3); For each of the clades the number of genera and species included in the analyses is given in brackets.

In all cases the monophyly of Trechina was strongly supported, with three main clades that were recovered in all analyses. Clade 1 (Figures 3a-c and 4) included most of the anisotopic species: the Pyrenean hypogean lineage (clade 1.1, Figures 3a and 4), the genus *Epaphius* and relatives (*Blemus* and some species of *Trechus*, clade 1.2) and a large group including most species of the genus *Trechus* plus a number of subterranean genera nested within it (clade 1.3). These included all the Alpine and Dinaric subterranean anisotopic genera with the only exception of *Doderotrechus*, plus some other genera from nearby areas (*Speotrechus*, *Jeannelius*, *Typhlotrechus*, *Sardaphaenops* and *Paraphaenops*, Figures 1 and 3).

Clade 2 (Figures 3d and 4), well-supported in all analyses (Bayesian posterior probability, pp = 1; ML bootstrap support, bt = 100), included all the isotopic species, with a largely paraphyletic *Duvalius* and the five subterranean genera (*Anophthalmus*, *Arctaphaenops*, *Agostinia*, *Luraphaenops* and *Trichaphaenops*) nested within it in uncertain positions. The Balkan genus *Pheggomisetes* was found sister to this clade 2, although with only moderated support (pp = 0.92/0.89; bt = 52). Clade 3, also well-supported (pp = 1; bt = 100) (Figures 3d and 4), included the remaining anisotopics: the Alpine genus *Doderotrechus* plus a species of *Trechus* from Tibet, *T. bastropi*. The isolated genus *Iberotrechus* was in some analyses recovered as sister to this clade, with low support.

#### Clade 1.1, the Pyrenean hypogean lineage

In agreement with previously published phylogenies [9,13], none of the three Pyrenean genera *Aphaenops*, *Geotrechus* and *Hydraphaenops* were recovered as monophyletic. The Pyrenean lineage was sister to the rest of clade 1 in all reconstructions, but this sister relationship was not well supported (Figures 3a and 4).

#### Clade 1.2, the “Epaphius” group

*Epaphius* and two species of *Trechus*, one widespread (*T. rubens*) and an Alpine endemic (*T. ovatus*), were grouped in a well-supported clade (pp = 1; bt = 100). We found the genus *Blemus* sister of this clade, although supported only in the Bayesian analyses (pp = 0.9; bt < 50).

#### Clade 1.3, Trechus and other hypogean anisotopics

This well-supported clade (pp = 1; bt = 88) was subdivided into two groups, the first (1.3.1) with Circum-Mediterranean species, gathering two Iberian *Trechus* with the highly modified genera *Sardaphaenops* (Sardinia), *Paraphaenops* (Iberia) and the Adriatic *Typhlotrechus*. This group was not well supported, although it was recovered both in the ML (bt < 50) and the Bayesian analyses (pp = 0.83/0.79). The second main group (1.3.2) was very well-supported (pp = 1; bt = 99) and included the Caucasian endemic genus *Jeannelius*, the French hypogean genus *Speotrechus*, a clade (1.3.2.1) with all the hypogean genera of anisotopic Trechina from the Alps (except *Doderotrechus*) plus the Dinaric hypogean genera, and a clade (1.3.2.2) with most of the *Trechus* species including many Alpine endemics and the type species of the genus, *T. quadristriatus*.

The clade 1.3.2.1 was well-supported (bt = 80; pp = 0.99/1) and included the subterranean genera *Orotrechus*, *Boldoriella*, *Lessinodytes*, *Allegrettia*, *Italaphaenops* and the two Dinaric genera *Adriaphaenops* and *Neotrechus* (Figure 3c). No epigean species were nested within this clade. The monophyly of both genera *Orotrechus* and *Boldoriella* was confirmed, whereas the position of *Lessinodytes*, *Allegrettia* and *Italaphaenops*, as well as that of the Dinaric species, was uncertain. A sister relationship was suggested in the ML analysis between *Allegrettia* and *Italaphaenops*, but without support and not recovered in the Bayesian analyses.

The sampled species of the genus *Orotrechus* were recovered as monophyletic, and divided in two groups of species: the first, poorly supported (pp = 0.54/0.97; bt = 66) including *O. vicentinus*, *O. fabianii*, *O. giordanii* and *O. montellensis*, and the second, well-supported (pp = 1; bt = 99), with all other sampled species (Figure 3c). The two aphaenopsian species *O. jamae* and *O. messai*, currently attributed to different species groups [20], were included in the same clade. The genus *Boldoriella* was found monophyletic with low support (pp = 0.86/0.92; bt = 54), but the separation of two subgenera within *Boldoriella* (*Boldoriella* and *Insubrites*[26]) was not supported by our analyses, as *B.* (*Insubrites*) *focarilei* was closer to *B.* (*Boldoriella*) *carminatii* than to *B.* (*Boldoriella*) *humeralis*.

The clade 1.3.2.2 was also well-supported (pp = 0.97/0.99; bt = 81), and included most of the species of *Trechus* sensu stricto (including the type species of the genus). Seven sub-clades were recovered with general good support, together with some isolated species of uncertain position (*T. karadenizus* and *T. lepontinus*, Figure 3b): (1) a Pyrenean-Cantabrian clade (pp = 1; bt = 77), which included 16 species, some of them hypogean (*T. beusti*, *T. navaricus*, *T. bouilloni*, *T. escalerae* and *Apoduvalius alberichae*); (2) the group of *T. fairmairei* and closely related species; (3) two species of the Maritime and Ligurian Alps, *T. liguricus* and *T. putzeysi*, as sister (with low support) to a species from the Eastern Alps, *T. gracilitarsis*; (4) the two species of the *T. strigipennis* species group as currently defined [14,39] (*T. pochoni* and *T. piazzolii*); (5) two species of *Apoduvalius*, with uncertain affinities; (6) the *Trechus fulvus* group, here represented by the species *T. fulvus* and *T. gigoni* (the latter described as *Antoinella*[40]); and (7) a clade containing *T. quadristriatus* and *T.obtusus*, plus some Alpine and Pyrenean endemics as well as species from Turkey.

#### Clade 2, the isotopic Trechina

The isotopic Trechina in the traditional sense [2,30] were confirmed as monophyletic with strong support (pp = 1; bt = 100), although the internal resolution was poor. The genus *Anophthalmus* was recovered as monophyletic (pp = 1; bt = 100), but depending on the phylogenetic method used it was sister to the rest of *Duvalius* species plus the subterranean genera *Agostinia*, *Trichaphaenops*, and *Arctaphaenops* (ML), or nested within this group (Bayesian), in both cases with low support (Figures 3d and 4). In all cases the rest of the subterranean isotopic genera were nested within a largely paraphyletic *Duvalius*. Some of the supported lineages had a well defined geographical distribution, such as a clade of species from the Maritime and Ligurian Alps, including the subterranean *Agostinia launi* plus the *Duvalius carantii*, *canevai*, *clairi* and *gentilei* species groups (sensu Vigna Taglianti [20]) (although with good support only in the Bayesian analyses, pp = 1/0.98; bt = 56); or *D. berthae* from Catalonia, unambiguously related to *D. raymondi* from Provence (Southern France) (pp = 1; bt = 94). Some other lineages corresponded to species groups defined on the basis of morphology, such as the subgenus *Euduvalius*, the *D. longhii* (pars) and *D. baldensis* groups sensu Vigna Taglianti [20], or the *D. microphthalmus* group sensu Jeannel [2].

#### Clade 3, Doderotrechus

The phylogenetic position of the genus *Doderotrechus* within Trechina was not well supported, although the Bayesian analyses suggested a closer relationship to the isotopics than to the main clade of anisotopics (Figures 3d and 4). *Doderotrechus casalei*, which was considered close to *D. crissolensis*[41], was recovered as sister to the rest of the species. The genus was found to be sister to a Himalayan *Trechus* recently described from south-central Tibet, *T. bastropi*, with strong support (pp = 1; bt = 100).

### Estimation of divergence dates

**Figure 5:**
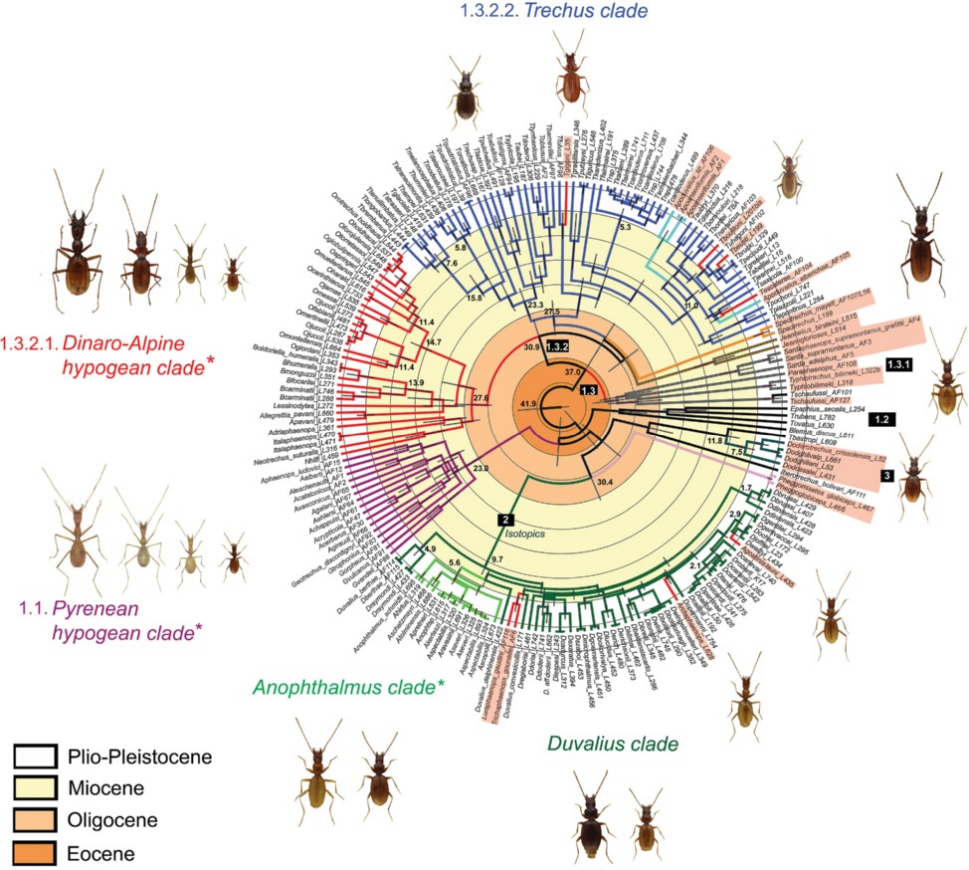
**Ultrametric time-calibrated tree obtained with BEAST for the combined dataset.** Grey bars, 95% confidence intervals of the estimated ages for the nodes. Hypogean clades and species are in red or identified by an asterisk.

According to our results, based on the rates estimated for a related ground beetle genus using the same genes [42], the origin of the Trechina radiation dates back to the middle Eocene (Figure 5). The origin of the main lineages within Trechina dates back to the late Eocene-early Oligocene, although the last common ancestor of the sampled species of some of them was estimated to be of much more recent origin, in the Miocene. Thus, the origin of the Pyrenean lineage was estimated to be at the end of Eocene, but the present-day species diversified from the early Miocene (c. 23 Ma, Figure 5). Similarly, the isotopic clade (clade 2) separated from the basal Trechina in the upper Oligocene, but the diversification of the group was estimated to be at the late Miocene, c. 10 Ma, in a rapid succession. Some of the subterranean clades (e.g. *Luraphaenops* + *Trichaphaenops* or *Anophthalmus*) originated short after the origin of the diversification of the clade, whereas the two other genera (*Arctaphaenops* and *Agostinia*) originated later, in the Lower Pliocene. The estimated age of the isolation between the only Iberian species, *D. berthae*, and the French *D. raymondi* was estimated to be at the end of the Miocene.

Other lineages had an earlier diversification, as estimated from our results. The hypogean Dinaric-Alpine clade (clade 1.3.2.1, Figure 3b) diversified in the late Oligocene, with an early isolation of the Dinaric species plus *Italaphaenops* from the other Alpine genera (although the phylogenetic position of *Italaphaenops* was not well supported, see above). The origin of the radiation including all the subterranean anisotopic genera but *Italaphaenops* was contemporaneous with that of the Pyrenean clade, in the early Miocene. Similarly, the diversification of the main clade of *Trechus* (clade 1.3.2.2) was also dated in the late Oligocene. Most of the subterranean colonisations within the *Trechus* lineage were also estimated to have occurred during the middle and late Miocene, as well as the separation between the Himalayan *Trechus bastropi* and the hypogean genus *Doderotrechus*. On the contrary, the subterranean genera *Sardaphaenops*, *Paraphaenops*, *Typhlotrechus*, *Speotrechus* and *Jeannelius* were estimated to be of Oligocene origin (Figure 5). The estimated age of the origin of the Sardinian endemic *Sardaphaenops* was 33 Ma.

## Discussion

### Phylogeny of the Alpine Trechina

In agreement with previous phylogenetic analyses [13,28,43] we found strong support for the monophyly of the subtribe Trechina, although the phylogenetic relationships among the main lineages of this subtribe remain uncertain. Contrary to the Pyrenean lineage, the Trechina of the Alps do not form a geographically well-defined unit, even when the widespread genera *Trechus* and *Duvalius* are not considered. Some genera of subterranean blind Trechini of the Dinarides (*Adriaphaenops* and *Neotrechus*, the two included in our study) were nested within the lineage of subterranean anisotopic Trechini from the Alps (our clade 1.3.2.1), stressing the need to include more Dinaric genera to establish the phylogenetic relationships of the group and understanding its origin and the colonisation of the area by subterranean Trechini. Recent discoveries of unexpected and remarkable genera in the Dinaric karst suggest that the subterranean fauna of this area needs further investigation [36-38].

Our age estimations were based on rates obtained in [42] for a related genus of groundbeetle using a combination of fossil and biogeograhic events (see Methods below), but were remarkably similar to those obtained by a previous work on western Mediterranean Trechini using as a calibration point the vicariant separation of the genus *Sardaphaenops* due to the tectonic drift of the Sardinian plate from the continent ca. 33 Ma [13]. Thus, even when the tectonic separation of Sardinia was not used as a calibration point in [42], our estimate for the origin of *Sardaphaenops* was 32.7 Ma, fully in agreement with a vicariant tectonic origin. Similarly, the estimated ages of e.g. the Pyrenean, *Duvalius* sensu *lato*, main group of *Trechus* and *Doderotrechus* + *Iberotrechus* crown clades were respectively 22.7, 9.6, 24.8 and 23.4 Ma in [13], versus 23.0, 9.7, 27.5 and 27.8 Ma here.

#### The anisotopic genera

Our results confirm the monophyly of the Pyrenean lineage [9], as none of the hypogean highly modified Trechini of the Alps and Dinaric chains were found to be related to it. The Pyrenean hypogean lineage remains isolated, with no clear affinities with other Trechini, and the similarities in the body shape of the species included in the *Aphaenops* lineage of Jeannel [2] should thus be the result of convergence or parallelism (see below). We also found support for the monophyly of the genus *Orotrechus*, characterised by the presence of a peculiar, synapomorphic structure of the lamellar parameres in the male genitalia and by having only the first tarsomere dilated in the male. These well defined morphological synapomorphies prevented authors from describing species with an aphaenopsian and non-aphaenopsian general body shape as different genera [2], as it happened in other cases. Thus, in the same genus it is possible to find all degrees of troglomorphism, from the small-sized, poorly specialised endogean species (such as *O. mandriolae* (Ganglbauer) or *O. cavallensis* Jeannel), to the anophthalmous hypogean species (such as *O. holdhausi* (Ganglbauer)) and finally the highly modified aphaenopsid species (such as *O. venetianus* (Winkler), *O. stephani* (J. Müller), *O. jamae*, *O. theresiae* Casale, Etonti & Giachino, *O. gigas* Vigna Taglianti and others). Our preliminary results suggest a complex history of the genus, with various species groups colonizing the same area at different times (e.g. several sympatric species with different degrees of troglomorphism are known in the Cansiglio-Cavallo massif in the Eastern Italian Alps [14]), as observed in the *Trechus* lineage. Although our sampling only included less than half of the described species, it is apparent that the genus needs a taxonomic revision, as none of the species groups previously suggested was found to be monophyletic [2,20,30,44,45]. We did not find, however, evidence for the assumed close relationship between this genus and *Neotrechus* (the “*Neotrechus* lineage” of Jeannel [2], accepted by all subsequent authors), although the lack of support does not allow to completely discard this hypothesis.

We also did not found a close relationship between the highly modified subterranean genera *Allegrettia*, from the Italian Alps, and *Jeannelius*, from the Caucasus, as hypothesized by some authors due to their strikingly similar external morphology [27,46,47]. The inclusion of other Caucasian genera will allow to test the alternative hypothesis of Jeannel [48], who suggested that *Jeannelius* is more related to other Caucasian genera than to the Italian *Allegrettia*.

An unexpected relationship was that of the western-Alpine genus *Doderotrechus* with a Himalayan *Trechus* from south-central Tibet. *Trechus bastropi* belongs to a group of species (the *T. dacatraianus* group) considered to be isolated within the Himalayan *Trechus* fauna owing to the structure of the male genitalia [31]. Such affinities were unexpected but not unique: in the western Alps there is a very isolated endemic subterranean species of Sphodrina ground beetle, *Sphodropsis ghilianii* (Schaum), the type species and only Alpine representative of a genus that includes several Asiatic species [49]. Furthermore, the presence in the Alps of other species of ground beetles considered to be relicts, belonging to genera mainly diversified in Asia (e.g. *Broscosoma* Rosenhauer [14]), is well known.

#### The isotopic genera

We found support for the monophyly of the isotopics, as a derived lineage nested within the anisotopics. Although the dichotomy isotopics-anisotopics is usually applied only to the species of Trechina [6], the asymmetry of the endophallus is a character present in all species of Trechini, and thus can be assumed to be plesiomorphic. According to our results, the diversification of the *Duvalius* clade occurred later than that of most of the anisotopics lineages, but was more geographically widespread and species rich than most of them. The possible relationship of the symmetry of the male genitalia with an increased diversification and geographical expansion seems difficult to explain from an evolutionary point of view, and due to its unique character it also cannot be statistically tested. The detailed study of the diversification of the isotopic lineage would require a more complete sampling of the high number of species of *Duvalius* from other geographical areas, from the Iberian peninsula and the Maghreb to central Asia. Similarly, the incomplete sampling of the species of *Anophthalmus* did not allow us to unambiguously solve if *Duvalius* (in its widest sense) is monophyletic and sister to it, or if *Anophthalmus* is a lineage nested within *Duvalius*, as previously hypothesized [2]. The sister relationship between *Anophthalmus* and the French *Duvalius delphinensis*, even if poorly supported and present only with Bayesian methods, is intriguing as this is considered to be an isolated species within *Duvalius*, but with some morphological characters common with *Anophthalmus* (shape of the male genitalia, pubescence of the head [2,23]). As seen with other genera, *Anophthalmus* includes species with various degrees of specialisation to the subterranean environment, from endogean or nivicolous to strictly troglobitic. The genus differs from *Duvalius* mainly in the elytral chaetotaxy [2], and previous attempts to clarify its taxonomy based on morphological characters have not reached a wide consensus (e.g. [50-54]).

The lack of resolution within the *Duvalius* lineage suggests a rapid diversification, which according to our estimations happened mostly in the late Miocene. The origin of some of the most troglomorphic genera, such as *Arctaphaenops*, *Agostinia, Luraphaenops* and *Trichaphaenops*, with a marked aphaenopsian habitus, was estimated to be Plio- or Pleistocene, more recent than that of other similarly modified genera from the area (e.g. *Italaphaenops* or *Lessinodytes*, from the early Miocene). The detailed phylogenetic affinities of these genera are still uncertain, although some of our results contradict assumed relationships. Thus, *Agostinia launi* appears to be closer to *Duvalius ochsi* and *D. gentilei* than to *D. carantii*, contrary to previous hypotheses [2,20]. *Trichaphaenops* and *Luraphaenops* were found to be sister taxa, suggesting a common origin of the morphological modifications of the two genera, but it would be necessary to study species of *Duvaliaphaenops* to further confirm this hypothesis. In all these cases the subterranean taxa tended to be related with other geographically close lineages, as found in previous works with subterranean Trechini and Leptodirini [9,10]. In agreement with this general pattern, our expectation is that the genus *Arctaphaenops* should also be closer to some *Duvalius* species of the same geographical area (which could not be included in our study) than to other aphaenopsian isotopic Trechini from the western Alps to which it was traditionally related (*Trichaphaenops*[6]). The large geographical gap between *Arctaphaenops* in the northeastern Alps and *Trichaphaenops* in the southwestern Alps and the Jura was explained by an hypothetical extinction of all intermediate forms by Pleistocene glaciers on the northern side of the Alps [6]. However, the ecology of some *Arctaphaenops* species, able to live at high altitude in cold, ice caves [55], contributes to weaken this hypothesis of an extinction by glacial episodes, not supported by our phylogeny. The inclusion of some of the northernmost species of *Trichaphaenops* and the easternmost *Duvalius* may help to clarify the phylogenetic relationships of *Arctaphaenops*.

Another example of unexpected affinities between geographically close taxa is that of the species *Duvalius raymondi* (French Provence) and *D. berthae* (Catalonia). *Duvalius berthae*, the only Iberian species of the genus, was thought to be very close to *D. lespesi*[2], from the French Causses. On the contrary, this later species was found to be related to *D. exaratus*, from the Eastern Alps, included in a different species group by Jeannel [2] (the *D. longhii* group) because it was pigmented and oculated. Vigna Taglianti [20] recognised the morphological peculiarities of *D. exaratus*, considering it an isolated species within the *D. longhii* group. Although not definitely discarded, the hypothesis of possible affinities between *Duvalius* and *Doderotrechus* previously suggested [13] was not supported by our results*.*

### Origin of the subterranean lineages of Alpine (and nearby areas) Trechini

Subterranean Trechini can be considered a good example of abiotically limited relicts [56,57]. Karstic areas, and more generally the deep soil fissures, may act as a buffer zone protecting subterranean animals from rapid and strong fluctuations of hygrometry, allowing the persistence of strict hygrophilous species with very reduced dispersal power outside this environment in the present day climate conditions. Subterranean species are known to be able to colonize all kinds of underground compartments [58-60], but, despite the physiological limitations, some amount of surface dispersal must have also occurred at some time in some taxa with already a high degree of troglomorphism, as shown by recent phylogenetic contributions [9,12,61]. The ancient origin of some of the subterranean lineages is in agreement with the age of many alpine caves (excluding paleokarst), which are of Pliocene or even Miocene age, although the karstification of some massifs colonised by hypogean Trechini (e.g. the Vercors massif in southeast France, the Lessini Mountains in northeast Italy, and others) has been more or less continuous since the Eocene [17].

Some of the discontinuities in the current distribution of the subterranean genera may also be explained by the geological history of the area. Thus, the geographical gap between the hypogean anisotopic Alpine and Dinaric genera may be explained by marine transgressions that occurred during the middle Miocene north of Zagreb [62], which could have led to the destruction of the fauna of this area and/or promoted the isolation of the ancestors of the extant species. It is interesting to note the presence in this area of the polytypic, enigmatic species *Typhlotrechus bilimeki*, the phylogenetic position of which was not well supported although our results suggested it may be related to the Iberian species *Trechus schaufussi* (“groupe *pandellei*” sensu Jeannel [2]), with an ancient (Oligocene) divergence. By the late middle Miocene the marine transgresion between the Dinarides and the Alps had definitively disappeared, and the ancestors of the isotopic genus *Anophthalmus* should have colonised the area.

According to our results, and in agreement with previous work of a more limited geographical scope [13], there are multiple origins of the highly modified hypogean Trechini in the western Palaearctic, with a high degree of convergence or parallelism among species occupying the same microenvironment. Both within the hypogean Alpine anisotopic and the Dinaric clades it is possible to recognise the same morphological types found in the Pyrenees [2,6,9]. Species occurring mostly in the soil, in superficial fissures or in the mesovoid shallow substratum (MSS), sometimes also nivicolous at high altitude, such as the genera *Orotrechus* and *Boldoriella*, have a similar general morphology than the species of *Geotrechus* in the Pyrenees, more stout and robust and with shorter legs and antennae. Highly hygrophilic, small species living in deep fissures and only exceptionally appearing in accessible caves, such as *Lessinodytes*, are very similar to the Pyrenean *Hydraphaenops*, with a narrow pronotum and head, often hairy body and long mandibles. Finally, species living in deep subterranean fissures and in natural or artificial caves (such as some *Orotrechus* and *Boldoriella, Allegrettia* and *Italaphaenops*) share with the Pyrenean *Aphaenops* an extreme elongation of the body, specially pronotum, head and appendages, leading to the typical “aphaenopsian” facies. It must be stressed that all three Pyrenean genera are para- or polyphyletic, increasing considerably the number of independent developments of each of the characteristic morphotypes [9].

**Figure 6:**
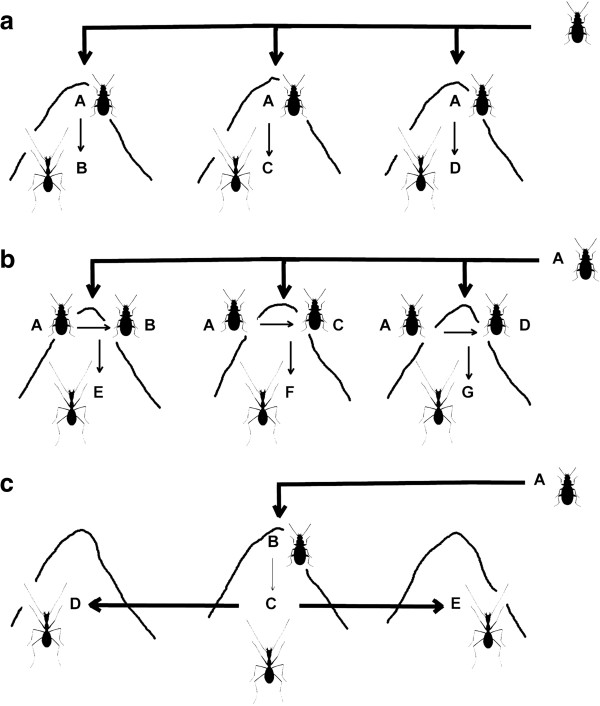
**Simplified representation of three idealised scenarios of subterranean colonization leading to an extant monophyletic hypogean lineage (see Discussion).** Different combinations of the three, in all possible degrees, can be found among the subterranean Trechini from the Alps. **a**: Three independent subterranean colonizations from a widespread epigean ancestor. **b**: Same as **(a)**, but preceded by speciation of the epigean ancestor. The three resulting epigean species colonize independently the subterranean medium. **c**: Single subterranean colonization, with subsequent dispersal and diversification of the hypogean species.

As noted above, in many cases the highly modified subterranean species can be related to other epigean (or less modified) taxa in the same geographical area, such as for example the species of the genera *Apoduvalius* and *Antoinella* (the latter already proposed as a synonym of *Trechus*, related to the species of the *fulvus* group [63]), which are confirmed as polyphyletic and to belong to the *Trechus* lineage. In the case of the *T. fulvus* group, it seems likely that from an epigean, widespread species (*T. fulvus*) there were many instances of independent populations colonising the subterranean environment (as schematised in Figure 6a), some of them recognised as distinct species but others only as subspecies or simple varieties, and most of them –but not all– wingless and with different degree of eye reduction [64]. This seems also to be the case of the widespread genus *Duvalius*, nested within which there are some highly troglomorphic species currently considered as distinct genera (*Arctaphaenops*, *Agostinia, Trichaphaenops* and *Luraphaenops*). In the *Duvalius* lineage, and contrary to what happens with the *T. fulvus* group, the origin of the highly troglomorphic taxa cannot be directly traced to a single, widespread species, so there is the possibility that the colonisation of the subterranean environment was preceded by local speciation of still epigean species (Figure 6b).

It is, however, remarkable that some monophyletic and exclusively subterranean lineages, without recent epigean relatives, are of ancient origin and include a high number of taxa in a relatively large geographical area, such as e.g. the Pyrenean lineage (clade 1.1 in Figure 4), the Dinaric-Alpine hypogean clade (clade 1.3.2.1) or the genus *Anophthalmus*. For these lineages, the most parsimonious scenario is a single origin of the subterranean habit and the associated troglomorphic characters (Figure 6c). This would imply the possibility that highly modified species were able to expand their geographical ranges and diversify, in some instances necessarily requiring surface displacements. This arguably happened in the highly modified Leptodirini genus *Troglocharinus* from the Pyrenees, which colonised the coastal area south of Barcelona during a climatically favourable time window in the early Pliocene [61]. In the case of the Dinaric-Alpine subterranean clade (1.3.2.1), which includes taxa from the Dinarides and the southeastern Italian Alps (i.e. the “Illyrian-Gardesan Dolomitic sector” sensu Ozenda & Borel [16]), the almost continuous presence of karst through this area (Additional file 1: Figure S1) may have allowed the displacement of species not able to leave the subterranean environment. The absence of subterranean anisotopic species in the northeastern calcareous Alps (Figure 1), in where there is only an isotopic subterranean genus of Trechini (*Arctaphaenops*, of a much more recent origin), would be in agreement with this hypothesis. Thus, it seems that no anisotopic taxa of this clade was able to cross the Suprapannonian sector [16,65], without karst in a large area around Graz (Additional file 1: Figure S1), suggesting that they were not able to disperse long distances outside the subterranean environment. It is always possible that the geographic expansion of this clade was due to epigean ancestors which are now all extinct or remain unknown, and that the troglomorphic species have thus an independent, local origin. However, at present there is no evidence to support this hypothesis, which on the other hand could never be falsified – only made unlikely.

## Conclusions

We have unveiled the complex history of a ground beetle lineage with multiple instances of independent colonisation of the subterranean world, showing that there is no simple, unique evolutionary pathway in which this could be achieved. Thus, among the Alpine fauna of Trechini we have recognised recent radiations with multiple origins of subterranean species (e.g. *Duvalius*, some lineages within *Trechus*); isolated, highly modified troglobitic genera of uncertain affinities (e.g. *Doderotrechus*, *Speotrechus*); and ancient, diverse lineages likely to have diversified and expanded once fully adapted to the underground life (the Dinaric-Alpine subterranean clade). It seems clear that the use of simplistic models may only lead to the artificial recognition of exclusive alternative hypotheses where there is a continuum of different processes. To understand the origin and evolution of the subterranean fauna it is thus necessary to comprehensively investigate the evidence provided by the phylogenetic and geographical context of each lineage individually.

## Methods

### Taxon sampling, DNA extraction and sequencing

Sampling was carried out in the Alps and nearby areas during 2011 and 2012. We included examples of all the endemic genera of hypogean Trechini from their entire known distribution area, with the only exception of *Aphaenopidius* and *Duvaliaphaenops*, of which no specimens could be obtained. Of the two Alpine genera with a widespread distribution (*Duvalius* and *Trechus*) numerous localities of hypogean and epigean species were sampled (Additional file 4: Figure S3; Additional file 5: Table S2). We also added some taxa from other geographical areas that have been related to the Alpine fauna (Pyrenees and Sardinia), plus two genera of hypogean Trechini from the Dinarides, one orophilous Himalayan species originally described as belonging to the genus *Trechus*, and a sample of the genera *Pheggomisetes* and *Jeannelius* from the Balkans and the Caucasus respectively*.* In total, we analysed 207 individuals of 173 species belonging to 31 genera (Additional file 5: Table S2, where authors and year of description of the studied species can also be found). Specimens were collected by hand or by means of pitfall traps containing propylene glycol, known to preserve DNA [66,67], and subsequently preserved in 96% ethanol. For some species we extracted DNA from dried specimens deposited in the ZSM. Extractions of single specimens were non-destructive, using the DNeasy Blood & Tissue Kit (Qiagen GmbH, Hilden, Germany). After extraction, specimens were mounted on cards and genitalia stored in water–soluble dimethyl hydantoin formaldehyde (DMHF) on transparent cards, pinned beneath the specimen. Vouchers and DNA aliquotes are stored in the collections of ZSM, IBE and MNHN*.*

The tree was rooted with a species of Patrobini (*Penetretus temporalis*), found to be sister to Trechinae (or Trechitae) [28,43]. In preliminary analyses other Trechini outgroups closer to Trechina (as e.g. Trechodina, *Perileptus* or *Thalassophilus*) were found to have extremely long branches and were not used to avoid analytical artifacts. The topologies obtained were, however, very similar (results not shown).

We amplified fragments of four mitochondrial genes: 3′ end of cytochrome c oxidase subunit (*cox1*) and a single fragment including the 3′ end of the large ribosomal unit (*rrnL*), the whole tRNA–Leu gene (*trnL*) and the 5′ end of the NADH dehydrogenase 1 (*nad1*). We also amplified two nuclear genes, an internal fragment of the large ribosomal unit 28S rRNA (*LSU*) and the 5′ end of the small ribosomal unit 18S rRNA (*SSU*) (see Additional file 6: Table S3 for the primers used). Sequences were assembled and edited using Sequencher TM 4.8 (Gene Codes, Inc., Ann Arbor, MI). Some sequences were obtained from Faille et al. [9,13] (Additional file 5: Table S2). New sequences have been deposited in the EMBL database (Accession Numbers: Additional file 5: Table S2).

### Phylogenetic analyses

We aligned the sequences using the MAFFT online v.6 with the Q-INS-i algorithm and default parameters [68]. Analyses were conducted on the combined data matrix with MrBayes 3.1.2 [69], using five partitions corresponding to the five genes used (the *trnL* and *rrnL* genes were included in the same partition). We used jModelTest 3.7 [70] to identify the best model of nucleotide substitution fitting each gene (partition). Two independent analyses of MrBayes ran until convergence using default values, saving trees each 5,000 generations. ‘Burn-in’ values and convergence were determined through the effective sample size (ESS) in Tracer v1.5 [71]. Results of the two runs were combined using LogCombiner v1.4.7 and consensus trees were compiled with TreeAnnotator v1.4.7 (Drummond & Rambaut 2007). Maximum likelihood analyses were conducted on the combined data matrix with RAxML GUI [72-74], using four partitions corresponding to the *cox1*, *rrnL + trnL + nad1, SSU* and *LSU* fragments with a GTR + I + G evolutionary model and default values for other parameters of the search [73].

### Estimation of divergence times

We used the Bayesian relaxed phylogenetic approach implemented in BEAST v1.7 to estimate the age of divergence of the different clades [72,75]. We pruned the outgroups and implemented a GTR + G model of DNA substitution to the same partition by genes used in the phylogenetic analyses. We used as priors the rates estimated for the same gene fragments in [42] for a related group of ground beetles (genus *Carabus*) based on a set of fossils and biogeographic events. We sequenced three fragments not included in [42], *nad1*, *trnL* and *SSU*, for which we used the same rates as *cox1*, *rrnL* and *LSU* respectively. The rates used were a strict clock for the mitochondrial genes, with mean rates of 0.0145 for the genes *nad1* and *cox1* and 0.0016 for the fragment *rrnL* + *trnL*; and a lognormal clock with mean rates of 0.0010 and 0.0013 for the *SSU* and *LSU* genes respectively. We used a Yule process of speciation as the tree prior, sampled the chain each 5,000 generations and used TRACER to determine convergence, measure the effective sample size of each parameter and calculate the mean and 95% highest posterior density interval for divergence times.

## Availability of supporting data

All supporting data are included in the Supplementary files with the exception of the sequences, deposited in the EMBL database with accession numbers HG514501-HG514996, and the alignments and trees, deposited in tree BASE: http://purl.org/phylo/treebase/phylows/study/TB2:S14674.

## Competing interest

The authors declare that they have no competing interest.

## Authors’ contributions

AF, AC and IR conceived the study. AF and AC coordinated the sampling. AF obtained the sequences. All authors contributed materials/reagents. AF and IR did the analyses and drafted the manuscript. All authors contributed to the writing and improving the manuscript, and approved the final version.

## Supplementary Material

Additional file 1: Figure S1 Map of the main alpine karsts (in black) (modified from [17]). Click here for file

Additional file 2: Figure S2 Lateral (a) and dorsal (b) views of a Trechini male genitalia, showing the anisotopic (1) and isotopic (2) positions of the copulatory piece (modified from [2]). Click here for file

Additional file 3: Table S1 List of the genera of Alpine Trechini, including number of species and previous hypotheses of relationships [2,11-13,20,22-25,27,30,46,47],[76-83]. Click here for file

Additional file 4: Figure S3 Map of the sampled localities. Click here for file

Additional file 5: Table S2 Material used in the study, with locality data, voucher number and accession numbers of the sequences. Click here for file

Additional file 6: Table S3 Primers used in the PCR amplification. Click here for file
