# Supplementary figures and images for: A molecular phylogeny of Alpine subterranean Trechini (Coleoptera: Carabidae)
Source: BMC Evol Biol. 2013 Nov 13;13:248. doi: 10.1186/1471-2148-13-248 (PMC3879191; doi:10.1186/1471-2148-13-248)

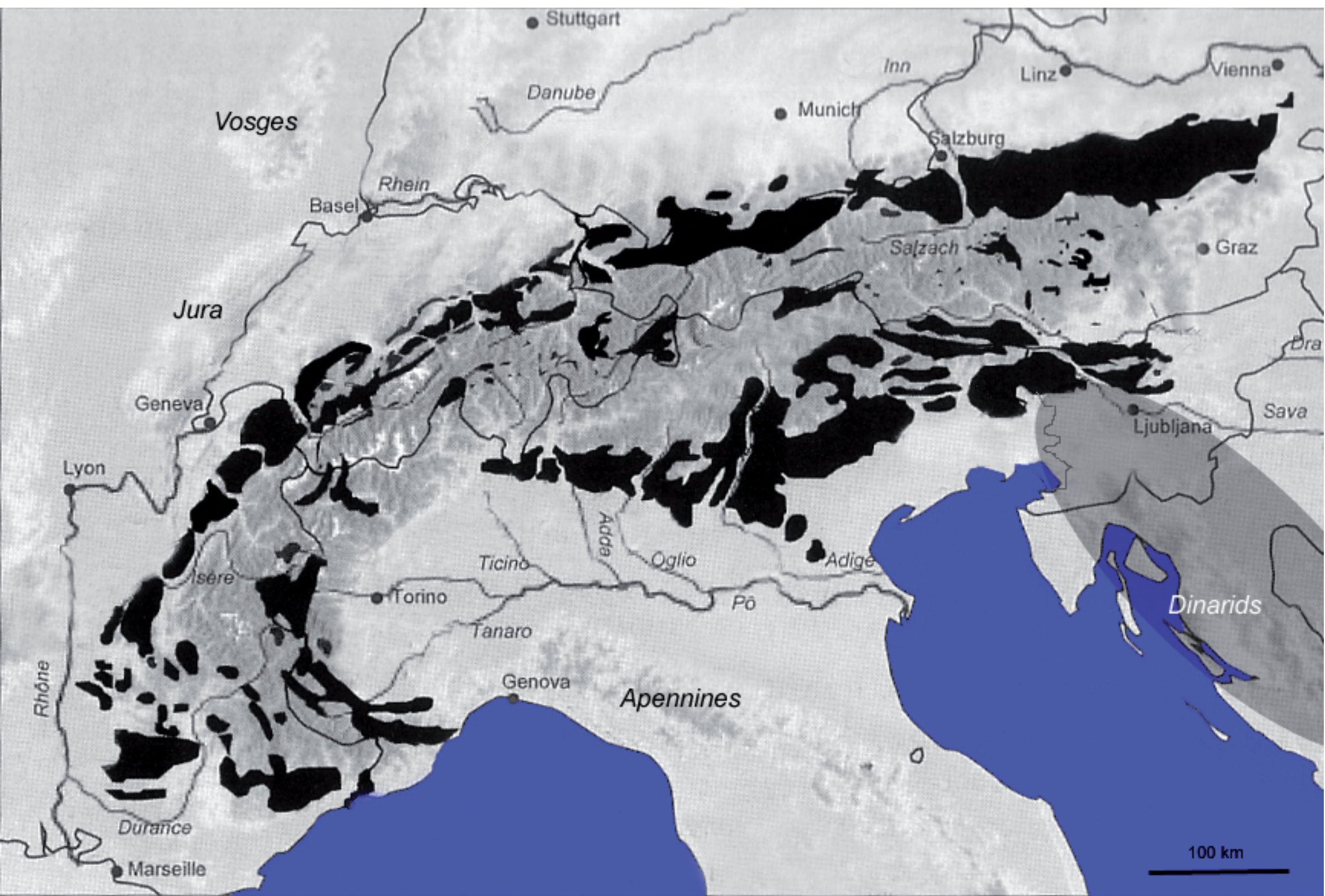

Supplement: Additional file 1: Figure S1 — Map of the main alpine karsts (in black) (modified from [17]). [file 1471-2148-13-248-S1.pdf]

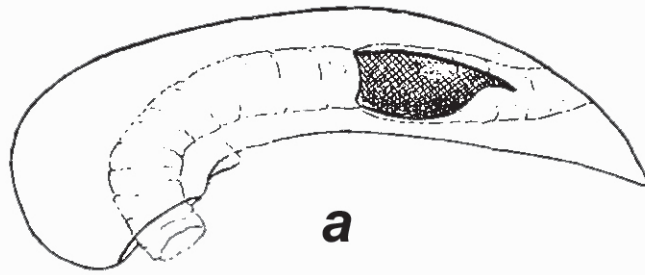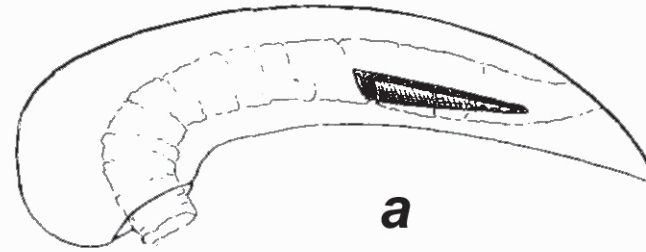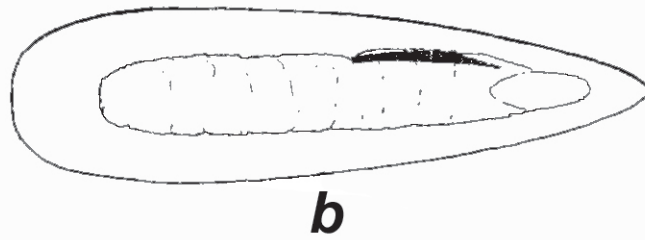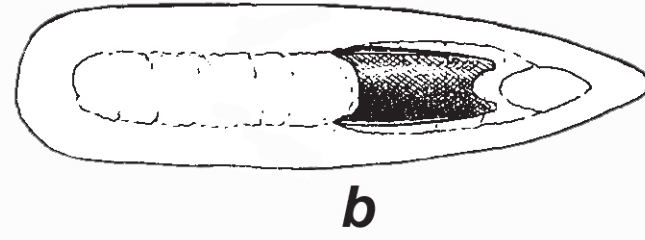

**1**

**2**

Supplement: Additional file 2: Figure S2 — Lateral (a) and dorsal (b) views of a Trechini male genitalia, showing the anisotopic (1) and isotopic (2) positions of the copulatory piece (modified from [2]). [file 1471-2148-13-248-S2.pdf]
