# Supplementary material for: A molecular phylogeny of Alpine subterranean Trechini (Coleoptera: Carabidae)
Source: BMC Evol Biol. 2013 Nov 13;13:248. doi: 10.1186/1471-2148-13-248 (PMC3879191; doi:10.1186/1471-2148-13-248)
Supplement: Additional file 3: Table S1 — List of the genera of Alpine Trechini, including number of species and previous hypotheses of relationships [2,11-13,20,22-25,27,30,46,47],[76-83]. [file 1471-2148-13-248-S3.doc]

**Additional file 4: Table S1.** List of the genera of Alpine Trechini, including number of species and previous hypotheses of relationships.

| **genus** | **author and publication date** | **species number** | **species included** | **distribution** | **previous hypotheses of relationships** |
| --- | --- | --- | --- | --- | --- |
| ***Anisotopics*** |  |  |  |  |  |
| *Allegrettia* | Jeannel, 1928 | 4 | 2 | Brescia area (Lombardy, Italy) | *Speotrechus* [2], *Jeannelius* [29, 30, 27], *Italaphaenops* [27], *Duvalius* [27] |
| *Boldoriella* | Jeannel, 1928 | 16 | 4 | Italian central pre-Alps | *Speotrechus* [2,32], *Doderotrechus* [13] |
| *Doderotrechus* | Vigna Taglianti, 1968 | 3 | 3 | Cottian Alps, Piedmont | *Typhlotrechus* [2], *Speotrechus* & *Boldoriella* [47, 76] |
| *Italaphaenops* | Ghidini, 1964 | 1 | 1 | Lessini Mountains [77,78] | *Arctaphaenops* [77], *Allegrettia* & *Orotrechus* [24,27] |
| *Lessinodytes* | Vigna Taglianti, 1982 | 3 | 1 | central pre-Alps from Brescia to Verona [25] | *Aphaenops*  phyletic lineage [13] |
| *Orotrechus* | J. Müller, 1913 | 37 | 14 | from the Garda lake  (Italy) to northeastern Slovenia | *Neotrechus* [2,13] |
| *Speotrechus* | Jeannel, 1922 | 1 | 1 | French Alps and the Cevennes [2, 22, 23] | *Paraphaenops* [2], *Trechus* [12] |
| *Trechus* | Clairville, 1806 | >800 | 59 | Mostly Palearctic region [11, 20] |  |
| *Typhlotrechus* | J. Müller, 1913 | 2 | 1 | Dinaric area (Croatia, Slovenia,  Bosnia Herzegovina and Italy near Trieste) | *Doderotrechus* [2] |
| ***Isotopics*** |  |  |  |  |  |
| *Agostinia* | Jeannel, 1928 | 1 | 1 | Marguareis and Mongioie massifs in the Ligurian-Maritime Alps | *Anophthalmus* [80], *Trichaphaenops* [2], *Duvalius* *carantii* group [2,13] |
| *Anophthalmus* | Sturm, 1844 | 48 | 11 | Slovenia, southern Austria, northeastern Italy and Croatia [11,20] |  |
| *Aphaenopidius* | J. Müller, 1909 | 2 | - | Austria, Slovenia | *Duvalius* and  *Anophthalmus* [2,81] |
| *Arctaphaenops* | Meixner, 1925 | 3 [11,83] | 1 | Austrian Alps | *Trichaphaenops* [2,82] |
| *Duvaliaphaenops* | Giordan, 1984 | 1 | - | French Maritime Alps | *Agostinia* [79] |
| *Duvalius* | Delarouzée, 1859 | >300 | 37 | widely distributed from Spain, Maghreb (Algeria) and  France in the west to central Asia and China in the East |  |
| *Luraphaenops* | Giordan, 1984 | 2 | 1 | Southeastern France | *Duvalius* [22] |
| *Trichaphaenops* | Jeannel, 1916 | 4 | 1 | Southeastern France | *Duvalius* [2,22], *Arctaphaenops* [2,82] |
