# Supplementary material for: A molecular phylogeny of Alpine subterranean Trechini (Coleoptera: Carabidae)
Source: BMC Evol Biol. 2013 Nov 13;13:248. doi: 10.1186/1471-2148-13-248 (PMC3879191; doi:10.1186/1471-2148-13-248)
Supplement: Additional file 4: Figure S3 — Map of the sampled localities. [file 1471-2148-13-248-S4.pdf]

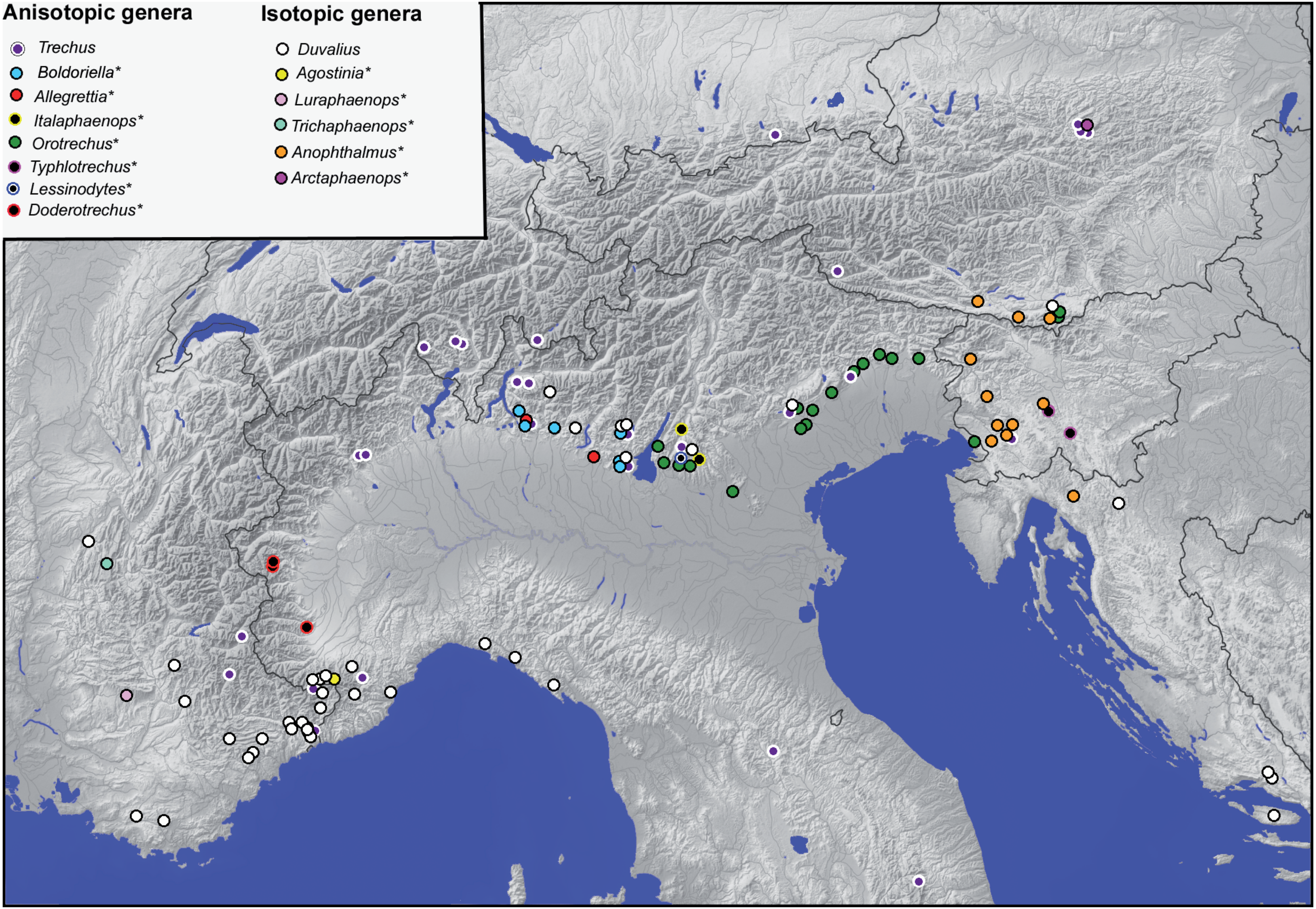
Anisotopic genera
Trechus
Boldoriella*
Allegrettia*
Italaphaenops*
Orotrechus*
Typhlotrechus*
Lessinodytes*
Doderotrechus*
Isotopic genera
Duvalius
Agostinia*
Luraphaenops*
Trichaphaenops*
Anophthalmus*
Arctaphaenops*
