# Supplementary material for: A molecular phylogeny of Alpine subterranean Trechini (Coleoptera: Carabidae)
Source: BMC Evol Biol. 2013 Nov 13;13:248. doi: 10.1186/1471-2148-13-248 (PMC3879191; doi:10.1186/1471-2148-13-248)
Supplement: Additional file 6: Table S3 — Primers used in the PCR amplification. [file 1471-2148-13-248-S6.pdf]

Additional file 6: Table S3. Primers used in the amplification.

Gene	Name	Sense	Sequence	Ref.
cox1	Jerry (M202)	F	CAACATTTATTTTGATTTTTTGG	[1]
	Pat (M70)	R	TCCA(A)TGCACTAATCTGCCATATTA	[1]
	Chy	F	T(A/T)GTAGCCCA(T/C)TTTCATTA(T/C)GT	[2]
	Tom	R	AC(A/G)TAATGAAA(A/G)TGGGCTAC(T/A)A	[2]
	Ron	F	GGATCACCTGATATAGCATTCCC	[1]
rrnL-nad1	16saR (M14)	F	CGCCTGTTTA(A/T)CAAAAACAT	[1]
	ND1A (M223)	R	GGTCCCTTACGAATTTGAATATATCCT	[1]
SSU	5’	F	GACAACCTGGTTGATCCTGCCAGT	[3]
	b5.0	R	TAACCGCAACAACTTTAAT	[3]
LSU	D1	F	GGGAGGAAAAGAAACTAAC	[4]
	D3	R	GCATAGTTCACCATCTTTC	[4]

References

1. Simon C, Frati F, Beckenbach A, Crespi B, Liu H, Flook P: **Evolution, weighting, and phylogenetic utility of mitochondrial gene-sequences and a compilation of conserved polymerase chain-reaction primers.** *Ann Entomol Soc Am* 1994, **87**, 651-701.
2. Ribera I, Fresneda J, Bucur R, Izquierdo A, Vogler AP, Salgado JM, Cieslak A: **Ancient origin of a Western Mediterranean radiation of subterranean beetles.** *BMC Evol Biol* 2010, **10**(29): 1-14. doi:10.1186/1471-2148-10-29
3. Shull VL, Vogler AP, Baker MD, Maddison DR, Hammond PM: **Sequence alignment of 18S ribosomal RNA and the basal relationships of adephagan beetles: evidence for monophyly of aquatic families and the placement of Trachypachidae.** *Systematic Biology* 2001, **50**: 945-969.
4. Ober KA: **Phylogenetic relationships of the carabid subfamily Harpalinae (Coleoptera) based on molecular sequence data.** *Mol Phylogenet Evol* 2002*,* **24**: 228-248.
